# A Rare Case of COVID-19 Infection Leading to Colonic Stricture: Case Report and Review of Literature

**DOI:** 10.7759/cureus.27043

**Published:** 2022-07-19

**Authors:** Komal Yousaf, Ali Toffaha, Ahmad L F Yasin, Noof Al Naimi, Ayman Ahmed, Mohamed Abu Nada, Mohammed Yousif, Amjad Parvaiz

**Affiliations:** 1 Department of Surgery, Hamad General Hospital, Doha, QAT; 2 Colorectal Surgery Unit, Department of Surgery, Hamad Medical Corporation, Doha, QAT; 3 Radiology, Hamad Medical Corporation, Doha, QAT

**Keywords:** colon, strictures, intestinal obstruction, constipation, covid-19, sars-cov-2

## Abstract

Coronavirus disease 2019 (COVID-19) predominantly targets the respiratory tract; despite gastrointestinal (GI) symptoms that may present in many patients, colonic strictures in coronavirus disease (COVID-19) patients are extremely rare and, to our knowledge, have never been reported. We, herein, present a case of a 59-year-old lady who developed intestinal obstruction due to colonic strictures shortly after recovering from complicated COVID-19 pneumonia. Ultimately, she underwent laparoscopic subtotal colectomy with ileorectal anastomosis. After a long recovery period, she was discharged in good status. It has been more than two years since COVID-19 was declared as a pandemic by the World Health Organization. Infected individuals have highly variable clinical manifestations, yet the pathogenesis, diagnosis and ideal management of each of these complications is not well described in literature.

## Introduction

The novel coronavirus disease 2019 (COVID-19) predominantly targets the respiratory tract; however, gastrointestinal (GI) symptoms may be present in up to 26% of infected individuals [1]. Furthermore, in about 10% of cases GI symptoms may present without respiratory manifestations. Despite these large numbers, GI involvement is unfortunately associated with an increase chance of delayed diagnosis in such patients [2]. 

Patients with digestive symptoms have a variety of manifestations with diarrhea, nausea or vomiting being the most common [2]. Several cases of severe GI involvement have also been published. Reports of cases such as bowel necrosis [3], acute bowel ischemia [4] and colonic gangrene with perforation [5] exist in the literature, however, almost all present as the primary manifestation of the infection with coexisting positive COVID-19 polymerase chain reaction (PCR) results. Moreover, colonic strictures in coronavirus disease (COVID-19) patients are extremely rare, and to our knowledge have never been reported.

In this article we report a case of a 59-year-old female patient, who presented with symptoms suggestive of progressive large bowel obstruction after complete recovery from COVID-19 pneumonia. Abdominal computed tomography (CT) scan showed evidence of colitis while the pathology of surgically resected colonic segment revealed the presence of strictures in the large bowel. We also employed a comprehensive literature on this rare condition.

## Case presentation

**Figure 1:**
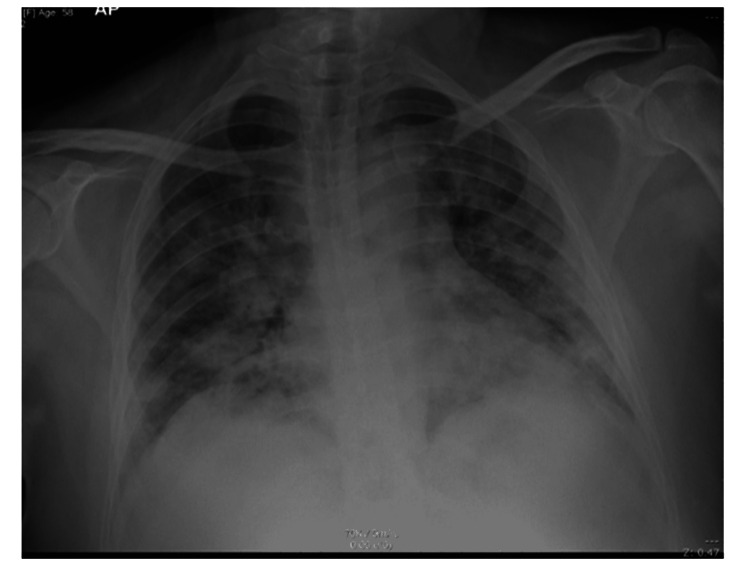
Frontal chest X-ray shows bilateral multiple confluent alveolar peripheral and medial peribronchovascular infiltrations with air bronchogram.

A 59-year-old lady with multiple comorbidities (diabetes, hypertension, dyslipidemia and hypothyroidism) presented to the emergency department (ED) with fever, and cough of two days duration. Investigations revealed a positive COVID-19 PCR. Her respiratory condition worsened; chest radiograph (Figure 1) revealed severe bilateral alveolar perihilar and peripheral infiltrations She was diagnosed with severe COVID-19 pneumonia and was shifted to the intensive care unit (ICU) for eight days where she required respiratory support including intubation and mechanical ventilation. She made a slow recovery resulting in step-down facility transfer and subsequently her discharge from the hospital. Three weeks later, she developed subglottic stenosis so she underwent micro-laryngoscopic carbon dioxide laser excision of the fibrotic tissue with balloon dilation.

**Figure 2:**
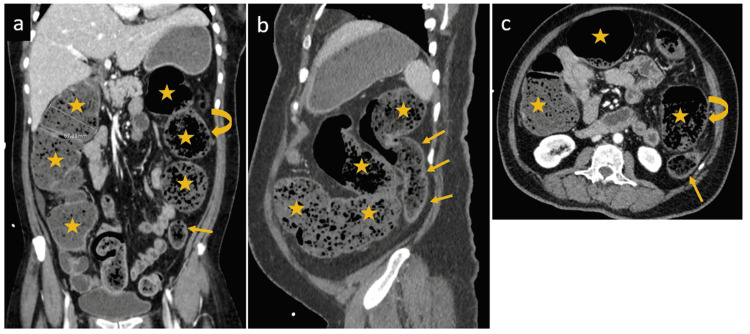
Initial contrast-enhanced abdominal CT scan. Coronal (A & B) and axial (C) images show diffuse dilation of the cecum, ascending and transverse colon (stars) up to the splenic flexure, followed by smooth tapering into the normal caliber descending and sigmoid colon (arrows) with the whole colon appearing impacted with feces. There is a short segment at the splenic flexure showing relative diffuse mild wall thickening with surrounding fat stranding (curved arrow)

A few days later she presented to the ED with symptoms of vomiting, severe generalized abdominal pain and constipation. The patient reported completely normal bowel habits before her COVID-19 infection. On examination, her temperature was 36.8 °C, heart rate was 78 beats per minute, respiratory rate was 27 breaths per minute and blood pressure was 90/60 mm Hg. She had a distended abdomen with generalized tenderness and bowel sounds were hypoactive. Digital rectal examination showed hard impacted stool. Investigations revealed raised white blood cell count of 17.0 x10^3/uL (4-10), low bicarbonate level, and mildly elevated C-reactive protein (CRP). CT scan of the abdomen showed diffuse dilation of the right colon and transverse colon up to the splenic flexure with smooth tapering towards descending colon the whole colon was impacted with feces and there was a short segment at the splenic flexure showing relative diffuse mild wall thickening with surrounding fat stranding likely represented a sign of colitis (Figure 2).

Bedside dis-impaction of faces was done and the patient improved and passed motion next day. After a few days of conservative management, she was discharged after complete resolution of her symptoms.

**Figure 3:**
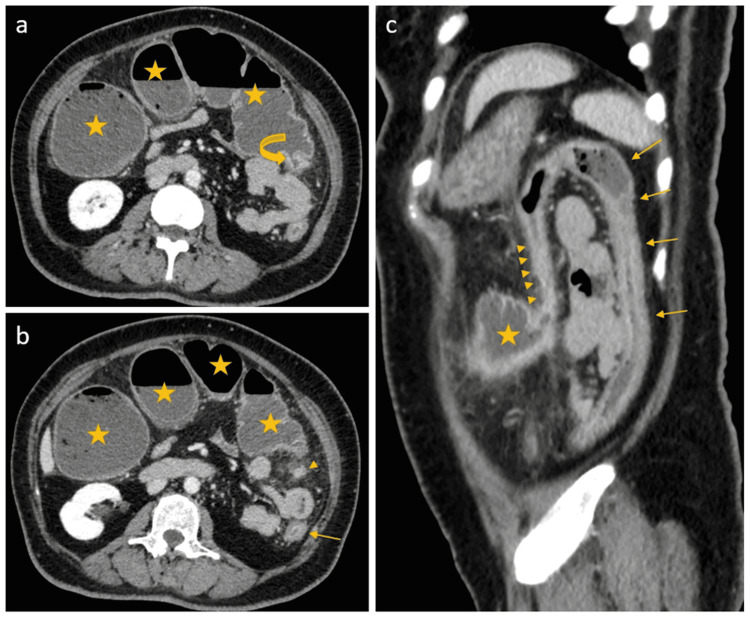
Interval contrast-enhanced abdominal CT scan. Axial (A & B) and sagittal (C) images show diffuse dilation of the ascending and transverse colon (star) with an abrupt cut off at the distal part of transverse colon showing a short segment of tightly narrowed hyperenhancing short segment of the splenic flexure (arrow heads) with surrounding fat stranding representing the transitional zone which is followed by collapsed distal colon (arrows) with faint surrounding fat stranding.

She was admitted again with similar complaints 26 days later. This time her abdominal CT scan showed diffusely dilated right colon and transverse colon with transitional zone was seen at splenic flexure followed by collapsed descending colon up to the anal canal. Moreover, the descending colon showed enhancing wall, mild peri-colonic fat stranding suggesting colitis (Figure 3). An urgent colonoscopy for possible stenting was attempted. It showed ulceration, friability and erythema of the sigmoid colon. There were multiple levels of narrowing starting 28 to 35cm from anal verge. Scope could not be passed beyond 35cm from the anal verge due to tight strictures. There was a clean based ulcer 4 cm from the anal verge with surrounding healed ulcer. Due to bowel friability, stenosis and risk of perforation, stenting was deemed unsafe. Differential diagnoses of ischemic bowel disease and COVID-19 colitis were made based on the results. Sample from sigmoid colon and rectal ulcer revealed mucosa with hyperplastic changes, fibrinopurulent exudate and granulation tissue consistent with adjacent ulcer. It was negative for viral inclusions. Biopsy from normal mucosa 28cm from anal verge showed no significant histopathologic abnormality.

Patient improved on conservative management and started passing stool and flatus. However, during her hospital stay she developed new onset stridor with exertion that resulted in difficulty sleeping at night. Fiberoptic bronchoscopy revealed severe subglottic stenosis and she was booked for dilatation and excision of fibrotic band. While being managed for the recurrent subglottic stenosis, she again developed symptoms of colonic obstruction and a nasogastric tube (NGT) was inserted. A decision for surgical resection of the involved colonic segments was discussed with the patient, however as a temporizing measure until managing subglottic stenosis, an urgent colonoscopy for decompression was done. It revealed a long ulcerated, friable segment 20 - 68cm from the anal verge with variable areas of narrowing, maximum at 68cm from anal verge which couldn’t be passed by 9.8mm scope due to tight stricture. Distal part of the sigmoid looked healthy. In the rectum, there was clean based ulcer 4cm from anal verge.

**Figure 4:**
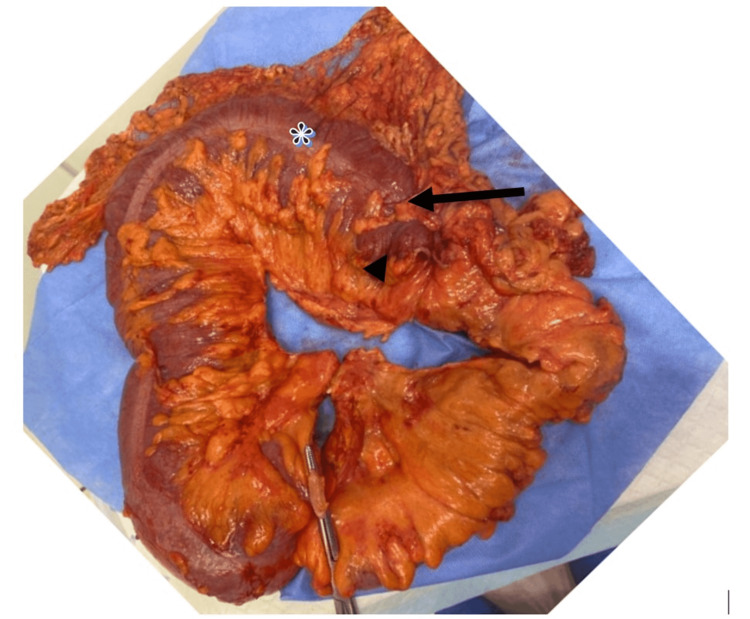
Resected colon with terminal ileum (bowel clamp), stricture (arrow), stapled resected attached small bowel (arrow head) and dilated transverse colon proximal to stricture (asterisk).

**Figure 5:**
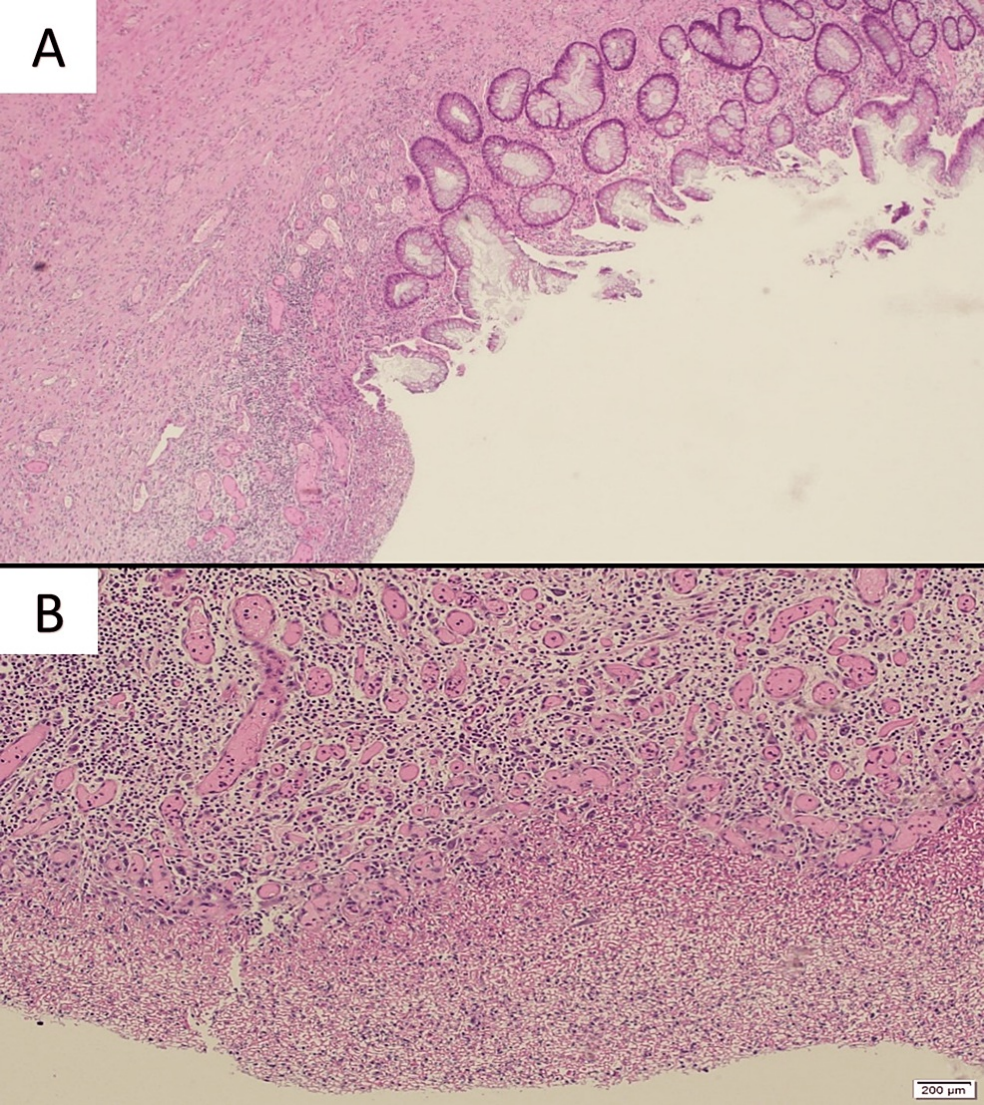
Histopathologic examination of resected specimen. A: Colonic mucosa with patchy ulceration with granulation tissue formation. The viable part of the mucosa shows mild crypt distortion. H&E,x4. B: Ulcerated colonic mucosa with inflamed granulation tissue. There are no granulomas or malignancy. H&E, x10.

After colonoscopy the patient started passing flatus and stool. Upon improvement of her symptoms, she underwent micro laryngoscopy, excision of subglottic tissue by punch forceps with subglottic balloon dilatation followed by mitomycin application. Recovery was smooth and her stridor resolved. Nine days later, laparoscopic subtotal colectomy with ileorectal anastomosis was undertaken for splenic flexure and descending colon strictures. Intra-operative findings showed a stricture at the splenic flexure with two small bowel loops attached to it (which were resected with the colon en-bloc) and a mucosal web in the distal rectum (Figure 4). Histologic examination of the resected part showed moderate active chronic colitis with ulceration, architectural distortion, granulation tissue formation with regenerative/ reactive atypia resulting in the colonic stricture at splenic flexure (Figure 5).

**Figure 6:**
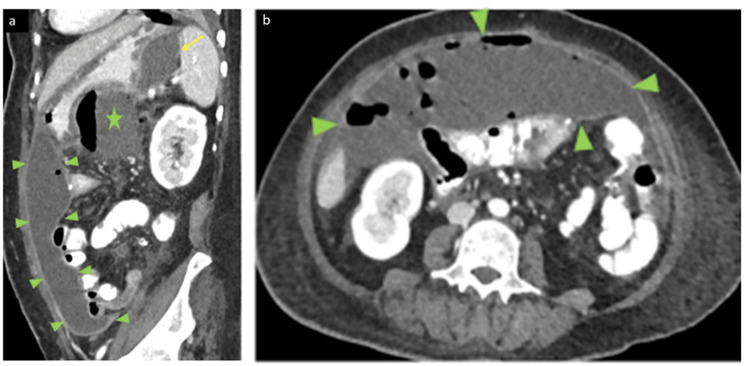
Postoperative contrasted abdominal CT scan Sagittal (a) and Axial (b) images show huge anterior abdominal peripherally enhancing fluid collection with gas bubble underlying the anterior abdominal wall (arrow heads) displacing the bowel posteriorly. Another two smaller collections also were noted along the gastro-splenic space (yellow arrow) and inferior to the stomach (star) abutting its greater curvature with the latter shows air fluid level.

Her postoperative course was complicated by non-ST elevation myocardial infarction (NSTEMI) that was managed medically. She also developed a septic shock and hypoxic respiratory failure for which she underwent an abdominal CT scan that showed multiple intra-abdominal collections (Figure 6). She was admitted to the intensive care unit and intubated. She was managed with ultrasound guided drainage of the collections, intravenous antibiotics and inotropes.

Patient then developed a large amount of rectal bleeding with hemoglobin drop. She underwent colonoscopy that showed an anastomotic site at 10cm and oozing blood from an ulcer at 5cm from the anal verge. Two endoclips were applied and haemostasias was achieved. She was stabilized after that, gradually weaned off ventilator support and transferred to the ward after a total stay of 30 days in the ICU.

She was further kept in ward for three weeks thereafter for completion of drainage of intrabdominal collections before being discharged from the hospital in good status.

## Discussion

Various theories have been published regarding the mechanism by which COVID-19 virus acts on the GI tract. Herberger et al. demonstrate the virus’ ability to cause direct cytotoxic effect on the enterocytes via angiotensin converting enzyme-2 (ACE-2) receptors [6]. Another proposed theory is ischemia secondary to microthrombi formation [7]. However, limited data is present on factors that contribute to a more severe response in the GI tract. Laski et al. described a case of pneumatosis intestinalis (PI) in a patient with COVID-19 who was on steroids for kidney transplant. They suggest that steroid intake prolongs the presence of COVID-19 virus in the GI tract, resulting in increased possibility of severe GI manifestation including PI [8]. Interestingly, there are increasing numbers of patients who developed airway strictures after intubation for COVID-19 infection [9], this may prompt further looking into the inflammatory response due to COVID-19 infection, as there might be specific pathways at the molecular level that induce excessive fibrotic and structuring response to COVID-19 infection, whether in the respiratory tract or GI as in our case.

The clinical presentation of our patient showed a progressive large bowel obstruction after complete recovery from COVID-19 pneumonia. Abdominal imaging revealed evidence of colitis and colonic stricture that were confirmed both surgically as well as on histopathological examination of the resected specimen. One case of GI involvement after apparent resolution of COVID-19 infection has been reported by Varshney et al. They reported a case of a 50-year-old lady who presented with features of large bowel obstruction five days after recovery from COVID-19 pneumonia. Contrast-enhanced CT of the abdomen and pelvis revealed grossly dilated distal segment of the descending colon and sigmoid colon with multiple diverticulosis. On laparotomy, the whole sigmoid colon was found to be gangrenous while the descending colon was ischemic with the presence of multiple perforations. Hartman’s procedure was performed; however, the patient rapidly deteriorated during postoperative recovery and expired the same day [5].

Although the exact pathogenesis of these events is not known, we suspect the persistence of viral load and ongoing infection within the GI tract may be the underlying cause. Both cases had a delay in diagnosis that cost the life of one patient and had significant morbidity to the other. We believe that factors such as negative nasopharyngeal PCR results and the lack of specific criteria for diagnosis of GI manifestations may have contributed to delay in the final diagnosis.

There should be an increased awareness and high index of suspicion among clinicians regarding the severe GI complications of COVID-19 and their occurrence during the post-recovery period to allow for timely diagnosis and prevent any further complications. We suggest that standard COVID-19 PCR from nasopharyngeal swab may be misleading in the case of GI involvement. Separate guidelines for diagnosis of GI disease may prevent cases of delayed diagnosis. Further, according to data published by Cheung et al., viral RNA persists in 50% of patients’ feces after recovery from respiratory infection [10]. We therefore recommend doing further studies to identify the significance of stool testing in patients with GI involvement before declaring them disease free from COVID-19 infection. Similarly, further research on factors contributing to more severe responses in gastrointestinal symptoms should be promoted.

## Conclusions

It has been more than two years since COVID-19 was declared a pandemic by the WHO. Infected individuals have highly variable clinical manifestations, including GI tract involvement, yet the pathogenesis, diagnosis and ideal management of each of these complications is not well described in literature. 

Clinicians should be more vigilant regarding the various possible complications that that COVID-19 patients may develop, so that they can proactively investigate and diagnose the rare and sometimes deleterious complications, including those affecting the GI tract.

Limited data is present on factors that contribute to a more severe response in the GI tract. Hence, further research should be promoted to help better allocate resources to those more vulnerable.
